# A Cytochrome P450 AaCP1 Is Required for Conidiation and Pathogenicity in the Tangerine Pathotype of *Alternaria alternata*

**DOI:** 10.3390/microorganisms13020343

**Published:** 2025-02-05

**Authors:** Huilan Fu, Wenge Li, Jintian Tang

**Affiliations:** 1 College of JunCao Science and Ecology, Fujian Agriculture and Forestry University, Fuzhou 350002, China; 2 Zhejiang Provincial Key Laboratory of Biometrology and Inspection & Quarantine, College of Life Sciences, China Jiliang University, Hangzhou 310018, China; liwenge0130@163.com (W.L.); jintiantang@cjlu.edu.cn (J.T.)

**Keywords:** *Alternaria alternata*, cytochrome P450, virulence, ACT toxin

## Abstract

Citrus *Alternaria* brown spot caused by the necrotrophic fungal pathogen of the tangerine pathotype of *Alternaria alternata* causes yield losses in global tangerine production. In this study, we focus on a cytochrome P450 monooxygenase encoding gene, *Aacp1*, for its role in the sporulation, toxin production, and virulence of the tangerine pathotype of *Alternaria alternata*. *Aacp1*-deficient mutants (*∆Aacp1*) produced significantly fewer conidia than the wild-type strain. Chemical assays demonstrated that *Aacp1* plays a negative role in resistance to oxidant stress and biosynthesis of ACT toxin. Virulence assays revealed that *ΔAacp1* fails to induce necrotic lesions on detached Hongjv leaves. Transcriptomic analyses of WT and *ΔAacp1* revealed that many metabolic process genes were regulated. Furthermore, our results revealed a previously unrecognized *Aacp1* affected the expression of the gene encoding a naphthalene dioxygenase (*AaNdo1*) for sporulation and full virulence. Overall, this study revealed the diverse functions of cytochrome P450 monooxygenase in the phytopathogenic fungus.

## 1. Introduction

Cytochrome P450s are heme-thiolate proteins and were discovered in 1958 [1]. The P450s regulate a broad range of physiological processes, such as drug metabolism, endogenous metabolism, environmental stress tolerance, and disease pathogenesis [2]. P450s are commonly distributed in all kingdoms and more than 728,000 sequences have been identified (https://www.uniprot.org/) through genome sequencing projects [3,4]. In humans, there are 57 functional genes in the P450 superfamily, which is classified into 18 families [5]. There are more than 85,000 genes classified into 805 families in fungi genomes, of which the CYP51 family is prominent [6]. Previous studies have shown that many of these genes have been identified as new families, indicating that the fungal genomes have a vast number of unexplored P450 genes with novel functions [7].

Cytochrome P450 monooxygenases play diverse biological functions in plant pathogens that contribute to full virulence. Furthermore, the expressions of an enormous number of P450 genes were significantly regulated during host–pathogen interactions, implying the P450s may participate in pathogenesis [7,8]. Previous research suggested that secondary metabolites are critical pathogenicity-related factors and regulated by P450s in plant pathogens. For example, the *VdCYP1* gene is required for the biosynthesis of 14 kinds of secondary metabolites and full virulence in *Verticillium dahlia* [9]. P450s contribute to the biodegradation of a vast array of toxic chemicals. In *Magnaporthe oryzae*, MoMCP1 plays a role in manganese detoxification and is required for pathogenicity [10]. P450s also participate in the biosynthesis of fungal membranes, for example, ergosterol biosynthesis in fungi [11]. Taken together, P450s play important roles in cellular metabolism, stress responses, secondary metabolites biosynthesis, and pathogenicity in phytopathogenic fungus.

The phytopathogenic fungus *A. alternata* causes diseases in over 100 plant species, such as citrus, tomato, apple, Japanese pear, and sunflowers [12]. Citrus *Alternaria* brown spot, a worldwide disease in tangerines, grapefruit, and their hybrids, which affects fruit yield and quality, is caused by the tangerine pathotype of *A. alternata* [13]. It was first identified in Australia and is widespread in citrus-producing countries in America, Africa, Asia, and Europe [14,15,16,17]. Investigating the pathogenesis mechanisms of the tangerine pathotype of *A. alternata* is crucial for developing rational and innovative control strategies.

Host-selective toxins (HSTs), such as the ACT toxin of the tangerine pathotype of *A. alternata*, HC toxin of *Helminthosporium carbonum*, CC toxin of *Corynespora cassicola*, and PC toxin of *Periconia circinata*, are low-molecular-weight secondary metabolites [18]. Electron micrographs of citrus revealed that susceptible citrus leaves treated with ACT toxin showed severe plasma-membrane invaginations and cell death, which suggests that ACT toxin acts as an important virulence factor in the tangerine pathotype of *A. alternata* [19]. The ACT toxin biosynthetic gene cluster is located in a small dispensable chromosome (CDC) and contains 25 genes [20,21]. Nine of these play an essential role in ACT toxin production and necrotic lesion formation [22,23,24,25,26,27,28]. Furthermore, the abilities to produce conidia, siderophores, and cutinases, and to detoxify reactive oxygen species (ROS), are also reported to be involved in the pathogenesis of the tangerine pathotype of *A. alternata* [29,30,31,32].

However, the function of P450s remain unclear in the tangerine pathotype of *A. alternata.* In this study, we investigated the function of a cytochrome P450 monooxygenase (Aacp1) in *A. alternata*. Our findings exhibited that *Aacp1* is required for sporulation, resistance to oxidative stress, production of ACT toxin, and full virulence. In addition, a naphthalene dioxygenase gene (*AaNdo1*) was identified to be regulated by *Aacp1* and markedly affects conidial production and virulence. These results revealed that *Aacp1* regulates the expression of *AaNdo1* for participation in sporulation, which was previously unrecognized. These findings provide novel insights into the role of cytochrome P450 monooxygenase in the tangerine pathotype of *A. alternata.*

## 2. Materials and Methods

### 2.1. Fungal Strain and Culture Conditions

The tangerine pathotype of *A. alternata* wild-type strain Z7 (CGMCC3.18907) from Zhejiang, China [21,33] was used as a parent strain for transformation throughout this study. Z7 and the resulting transformants (*ΔAacp1*, *Aacp1-c*, and *ΔAaNdo1*) were cultured at 26 °C on potato dextrose agar (PDA) or in potato dextrose broth (PBD) (Hopebio, Qingdao, China) in a shaker set at 160 rpm in the dark. Conidia were collected from fungal cultures incubated in the light for 7 days. All fungal strains used in this study were preserved in 15% glycerol solutions stored at −80 °C.

### 2.2. Generation of Aacp1 Deletion and Complementation Mutants

The *Aacp1* deletion mutants were created using the protocols as previously described [31]. In brief, a double-joint PCR was used to generate three hybrid DNA fragments. An upstream fragment (696 bp) and a downstream fragment (614 bp) of the *Aacp1* gene were independently amplified from the Z7 genome using the primers listed in Supplementary Table S1 and fused with a bacterial phosphotransferase B gene (*HPH*). The fusion fragments were transformed into protoplasts prepared from Z7 with polyethylene glycol (PEG) (Sigma-Aldrich, St. Louis, MO, USA) and CaCl_2_ (Guoyao, Shanghai, China) [34]. Putative transformants were picked from PDA containing 100 μg/mL hygromycin (Roche Applied Science, Indianapolis, IN, USA), verified by PCR assays with specific primers (Supplementary Table S1).

For complementation of the mutant, the full-length *Aacp1* gene including its promoter sequences (1166 bp) and 3’-UTR sequences (323 bp) was amplified and cloned onto the p1300-NEO plasmid [35]. The recombined plasmid was transformed into the protoplasts prepared from a *ΔAacp1* mutant. Putative complementation transformants were picked up from PDA containing 100 μg/mL G418 (Sangon Biotech, Shanghai, China), verified by PCR assays with relevant primers (Supplementary Table S1).

### 2.3. Phenotypic Analysis

For conidiation assays, the fungal strains were inoculated on V8 juice agar for 7 days. Spore germination assay was performed by spreading 50 μL spore suspension (conc. 1 × 10^5^ spore/mL) of the strain on a water agar (WA) plate at 26 °C for 12 h. The germination of spores (n = 300) was observed under a microscope. Three biological replicates were used for each strain and each experiment was repeated three times independently. The significance of treatments was determined by analysis of variance, and treatment means were separated by Ducan’s *t*-test (*p* ≤ 0.05).

For vegetative growth assays, fungal strains were cultured on potato dextrose agar (PDA) medium for 2 days. For assays of stress tolerance, fungal mycelia were transferred onto PDA amended with oxidants, cell wall-interfering agents, or other indicated chemicals [31]. Fungal radial growth was determined 4 days after incubation. Compounds used for sensitivity tests included hydrogen peroxide (H_2_O_2_), diethyl maleate (DEM), cumyl hydroperoxide (CHP), tert-butyl-hydroxyperoxide (T-BHP), sodium dodecylsulfate, Congo red, Sorbital, and NaCl. The size of colonies was measured using Image J software (https://imagej.net/ij/).

### 2.4. ACT Toxin Assays

To determine the ACT toxin production, each strain was grown in modified Richards’ solution at 26 °C for 24 d in a shaker (100 rpm) in the dark [36]. Culture filtrates after passing through filter paper were mixed with 3 mL Amberlite XAD-2 resins (Sigma-Aldrich) and incubated for 1 h. Amberlite XAD-2 resins were collected by passing through a filter paper and ACT toxin was dissolved in 13 mL methanol. ACT toxin was extracted for HPLC examination according to the previously reported protocol [37]. All tests were performed at least three times, each with three replicates.

ACT was separated in a Diamonsil Plus 5um C18 column (4.6 × 250 mm) attached to a Waters 880-PU HPLC system using methanol: Milli-Q (MQ) water: acetic acid (60:40:1, *v*/*v*) solution as a mobile phase at a flow rate of 1 mL/min. ACT was detected by a UV detector (Waters^TM^, Milford, MA, USA) with absorbance set at 290 nm. The percentage of ACT toxin reduction was calculated by dividing the comparative difference of the peak areas of retention time at 6.9 min by the wild-type areas and multiplying by 100.

### 2.5. Pathogenicity Assays

The virulence of each Aa. strain on detached Hongjv leaves was assayed as described previously [38]. In brief, 5 mm mycelial plugs of each strain taken from the edge of a 3-day-old colony were inoculated on the top of detached citrus (*Citrus reticulata*, cv. Hongjv) leaves. After inoculation, the leaves were placed in a closed plastic box at 26 °C for 3 days for the infection. The size of necrotic lesions was measured using Image J software. The experiment was repeated three times, each with three replicates.

### 2.6. RNA-Seq and Real-Time PCR Analysis

For RNA-seq and RT-qPCR assays, the mycelia of tested strain were cultured in PDB for 2 days, harvested, and ground in liquid nitrogen for RNA isolation. Total RNA was extracted using the Axygen RNA purification kit (Capital Scientific, Union City, CA, USA) and determined with a Nanodrop 8000 spectrophotometer. The samples were sequenced on DNBSEQ-T7. An aliquot of 5 μg of total RNA was retro-transcribed to cDNA using HiScript III RT SuperMix for qPCR (Vazyme, Nanjing, China). The relative transcript levels of genes were determined by quantitative real-time PCR in a CFX96 real-time system (Biorad, Hercules, CA, USA) with the following cycling conditions: 95 °C/30 s and then 40 cycles of 95 °C/10 s, 60 °C/30 s. The β-actin coding gene was used as a reference and each experiment was repeated three times. ClusterProfiler software (4.14.4) was used to test the statistical enrichment of DEGs in the KEGG pathways and Gene Ontology (GO).

Expression of *Aacp1* in the wild-type strain was inoculated to detached Hongjv leaves, purified at 30min, 12h, and 24h after inoculation, and used for cDNA synthesis and qRT-PCR analysis.

### 2.7. Statistical Analysis

Statistical comparisons were performed using SPSS statistics 19 (IBM, Armonk, NY, USA) and GraphPad Prism 5. All data are presented as means ± standard deviation. The significance of the results was assessed using independent sample *t*-tests or one-way ANOVA, with post hoc comparisons performed using Tukey’s Honest Significant Difference (HSD) test (*p* < 0.05).

## 3. Results

### 3.1. Identification and Deletion of Aacp1

The sequence of the *Aacp1* gene (*AaCP1*, PQ559313) encoding a cytochrome P450 monooxygenase was obtained from the complete genome sequence of the tangerine pathotype of *A. alternata* wild-type strain Z7 (WT)(GCA 001572055.1). The *Aacp1* gene was found to contain a 1630 bp open reading frame interrupted by a small intron of 49 bp, which could be translated into a protein with 527 amino acids. The phylogenetic tree revealed that Aaprb1 shared strong identities with the cytochrome P450 monooxygenase of different fungi species (Figure S1A).

To investigate the function of *Aacp1*, we generated deletion mutants using the homology recombination strategy, as illustrated in Figure S1B. Candidate mutants were identified by PCR and successful disruption of *Aacp1* in the two mutants *ΔAacp1-1* and *ΔAacp1-2* (Figure S1C). Since the two disrupted mutants were similar in terms of colony appearance, conidial production, and virulence, *ΔAacp1-1* was used in further study (*ΔAacp1*). A complementation strain designated *Aacp1-c* was also examined by PCR (Figure S1C).

### 3.2. Aacp1 Is Required for Conidial Production

**Figure 1:**
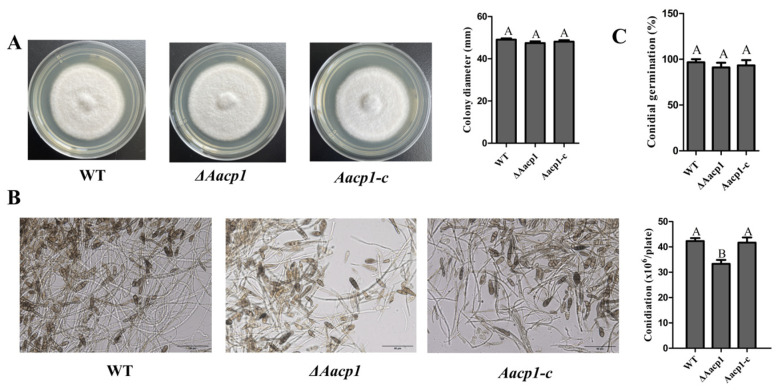
*Aacp1* is required for spore formation. (**A**) Vegetative growth of *A. alternata* WT *ΔAacp1*, and *Aacp1-c* on potato dextrose agar (PDA) medium at 48 h. (**B**) Conidiation of WT, *ΔAacp1*, and *Aacp1-c* on V8 juice agar and cultured at 26 °C for 7 days. (**C**) Conidial germination rate of WT, *ΔAacp1*, and *Aacp1-c* strains. Conidial suspensions were sprayed on water agar and incubated at 26 °C for 12 h. Different letters indicate significant differences at *p*-value < 0.05.

On V8 juice medium, conidial production in *ΔAacp1* reduced by as much as 21% compared with wild type (WT) (Figure 1B). Sporulation was restored to the WT level in the *Aacp1-c* strain (Figure 1B). *ΔAacp1* strains showed similar germination rates of spores at levels comparable to those of WT and *Aacp1-c* (Figure 1C). However, there were no significant differences between WT and *ΔAacp1* in the hyphal growth (Figure 1A).

### 3.3. Aacp1 Is Involved in Resistance to Oxidative Stress Tolerance

**Figure 2:**
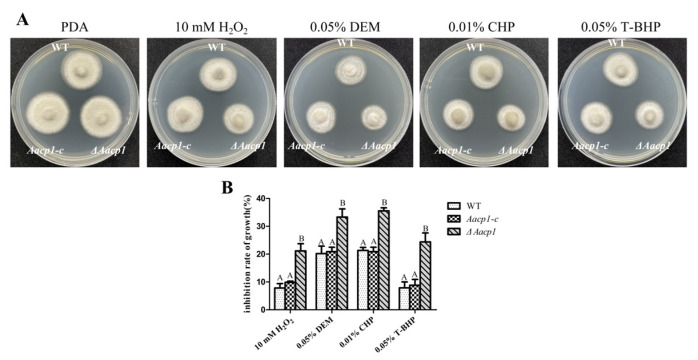
*Aacp1* is required for oxidative stress resistance. (**A**) Sensitivity tests were conducted by growing fungal strains on PDA amended with 10 mM hydrogen peroxide (H_2_O_2_), 0.05% diethyl maleate (DEM), 0.01% cumyl hydroperoxide (CHP), and 0.05% tert-butyl-hydroxyperoxide (T-BHP). (**B**) Growth inhibition rates of the strains cultured on PDA plates containing 10 mM H_2_O_2_, 0.05% DEM, 0.01% CHP, and 0.05% T-BHP. Means indicated by the same letter in a panel are not significantly different from one another, *p* < 0.05.

Chemical sensitivity assays revealed that *ΔAacp1* was sensitive to oxidizing agents. Compared to the WT, *ΔAacp1* showed significant difference in growth inhibition on PDA amended with hydrogen peroxide (H_2_O_2_), diethyl maleate (DEM), cumyl hydroperoxide (CHP), and tert-butyl-hydroxyperoxide (T-BHP) (Figure 2). As assayed on PDA, *ΔAacp1* did not change sensitivity to sodium dodecylsulfate, Congo red, Sorbital, or NaCl (Figure S2).

### 3.4. Aacp1 Is Required for Lesion Formation and ACT Toxin Biosynthesis

**Figure 3:**
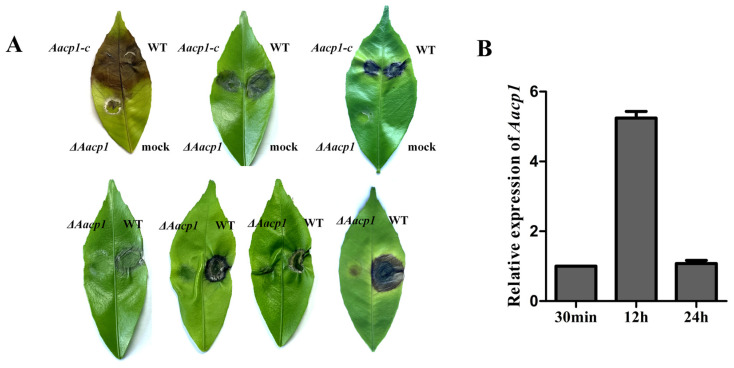
*Aacp1* is required for *Alternaria alternata* pathogenesis to citrus leaves. (**A**) Pathogenicity test of *ΔAacp1* on detached inoculated leaves. WT, *ΔAacp1*, and *Aacp1-c* were inoculated onto detached leaves of Hongjv by placing mycelial plugs and maintaining in a moisture plastic box for lesion development. (**B**) The expression of *Aacp1* in the wild-type strain inoculated to detached Hongjv leaves. RNA was purified at 30 min, 12 h, and 24 h after inoculation, and used for cDNA synthesis and qRT-PCR analysis.

Quantitative RT-PCR (qRT-PCR) analysis showed that the expression of *Aacp1* in the WT was upregulated as much as 5-fold 12 h post-inoculation (hpi) (Figure 3B), implying *Aacp1* may participate in pathogenesis. Fungal infection tests using mycelial plugs revealed that *ΔAacp1* failed to produce necrotic lesions on detached Hongjv leaves three days post-inoculation (dpi) (Figure 3A). Significantly necrotic lesions were observed on Hongjv leaves inoculated with WT and *Aacp1-c* 3 dpi (Figure 3A).

**Figure 4:**
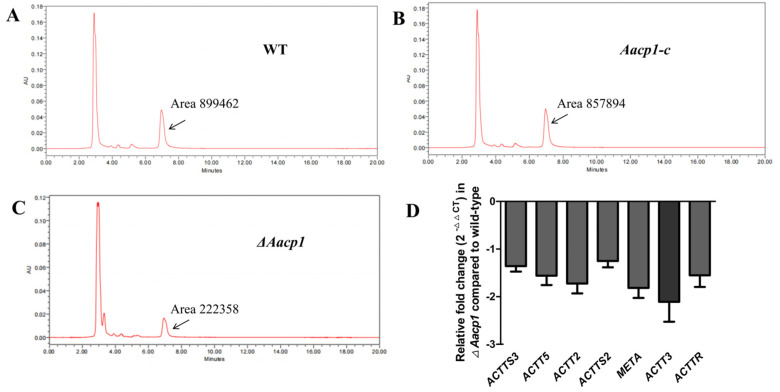
*Aacp1* is required for ACT toxin biosynthesis. (**A**–**C**) HPLC analysis of ACT toxin purified from culture filtrates of wild type, *Aacp1-c*, and *ΔAacp1*. The peak representing ACT toxin is indicated by a black arrow. (**D**) The relative expression level of the ACT biosynthetic genes in the WT and the *ΔAacp1* strains. The *β*-actin coding gene was used as the reference gene.

The WT, *ΔAacp1*, and *ΔAacp1* were cultured in Richard’s medium for 24 days for ACT toxin production. Samples prepared from culture filtrates of the fungal strains and analyzed by HPLC revealed significant differences in the level of toxin production. *ΔAacp1* reduced toxin production by 75% compared to the WT and *ΔAacp1*, based on peak areas (Figure 4A–C). Expression levels of *ACTTS3*, *ACTT5*, *ACTT2*, *ACTTS2*, *META*, *ACTT2*, and *ACTTR* were downregulated in *ΔAacp1* compared to the WT (Figure 4D).

### 3.5. Aacp1 Regulates Metabolic Process of the Tangerine Pathotype of A. alternata

**Figure 5:**
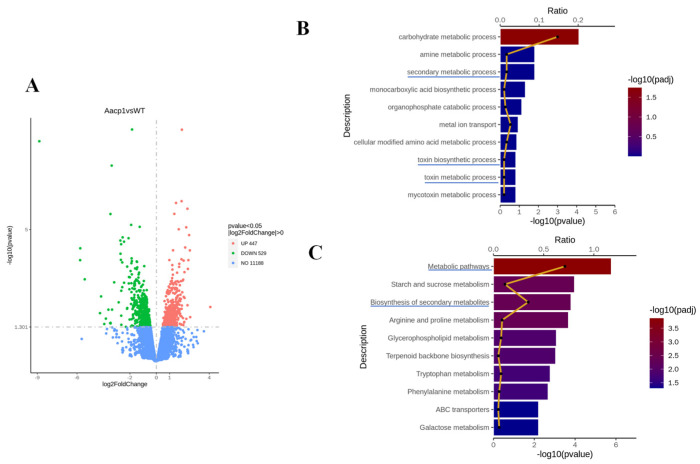
*Aacp1* regulates the metabolic process of *A. alternata.* (**A**) Volcano plot of transcriptome data. (**B**) GO enrichment of biological processes in which differentially expressed genes (DEGs) were involved. (**C**) Downregulation of KEGG pathways in WT and *ΔAacp1*.

Transcriptomic analysis was performed to compare the differentially expressed genes between *ΔAacp1* and the wild type during hypha growth. In total, 976 genes showed significant differential expression in the *ΔAacp1* strain, characterized by |log2foldchange| > 0 and *p*-values < 0.05. A total of 447 genes were upregulated and 529 genes were downregulated in *ΔAacp1* compared with the WT (Figure 5A). Gene Ontology (GO) enrichment and Kyoto Encyclopedia of Genes and Genomes (KEGG) pathway analysis were used to investigate the potential functions of the DEGs. GO enrichment revealed that the genes relating to the metabolic process were regulated in the *ΔAacp1* mutant, including the carbohydrate metabolic process, amine metabolic process, and secondary metabolic process (Figure 5B). According to the KEGG analysis, expression of the genes associated with metabolic pathways, starch and sucrose metabolism, and biosynthesis of secondary metabolites was significantly decreased in *ΔAacp1* (Figure 5C).

### 3.6. Aacp1 Is Involved in Pathogenesis Partially by Its Regulating AaNdo1

**Figure 6:**
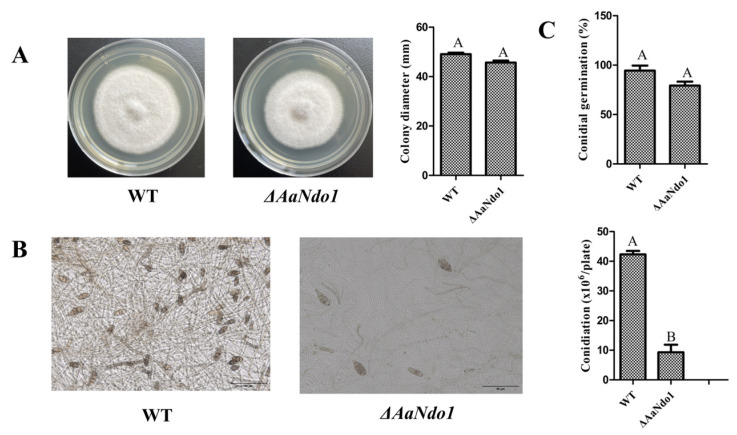
Characteristics of *AaNdo1* deficiency mutants. (**A**) Vegetative growth of *A. alternata* WT *ΔAaNdo1* on potato dextrose agar (PDA) medium at 48 h. (**B**) Conidiation of WT and *AaNdo1* on V8 juice agar and cultured at 26 °C for 7 days. (**C**) Conidial germination rate of WT and *AaNdo1* strains. Conidial suspensions were sprayed on water agar and incubated at 26 °C for 12 h. Different letters indicate significant differences at *p*-value *<* 0.05.

**Figure 7:**
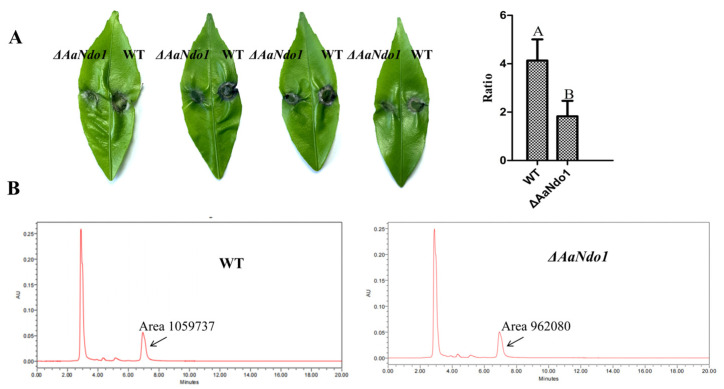
AaNdo1 is required for *Alternaria alternata* pathogenesis. (**A**) Virulence assays on citrus. Hongjv leaves were inoculated by placing mycelial mass prepared from WT and *ΔAaNdo1* strains and maintained in a moisture plastic box for lesion development. The bottom panels are the percent changes in necrotic lesions of the leaves calculated in relation to the leaf area, which is also indicated. Different letters indicate significant differences at *p*-value < 0.05. (**B**) HPLC analysis of ACT toxin purified from culture filtrates of WT and *ΔAaNdo1*. The peak representing ACT toxin is indicated by an arrow.

Analyses of RNA samples by transcriptome analysis and qRT-PCR revealed that the expression of the *AaNdo1* gene encoding a naphthalene dioxygenase was decreased in *ΔAacp1* (Figure S3A). To determine if *AaNdo1* is involved in virulence in *A. alternata*, the *AaNdo1* gene was disrupted using a homologous recombination in the wild-type strain and deletion transformants were identified by PCR (Figure S3B). There was no significant difference in fungal growth, conidia germination, or ACT toxin production of the WT and *ΔAaNdo1* (Figure 6A,B and Figure 7B). Virulence tests on detached Hongjv leaves with mycelial mass revealed that *ΔAaNdo1* could induce significantly smaller necrotic lesions compared to those induced by the WT at 3 dpi (Figure 7A). Noticeably, sporulation reduction in *ΔAaNdo1* was more drastic, reducing by as much as 75% compared to the WT after 7 days of incubation on V8 juice medium (Figure 6B). The transcript levels of other genes associated with sporulation were similar among the WT and *ΔAacp1* (Table S2).

## 4. Discussion

Cytochrome P450 monooxygenases are involved in a wide range of metabolic pathways in all living cells. Fungi utilize the P450s to achieve successful infection in the plant host by producing secondary metabolites, resistance to iron, drug, and oxidative stress, and inhibiting plant immune responses [39]. Therefore, investigation of cytochrome P450 would expand our understanding of the pathogenesis mechanisms of the tangerine pathotype of *A. alternata*. In this study, we explored the function of a cytochrome P450 gene, *Aacp1*, and performed transcriptome analysis of *A. alternata* WT and *ΔAacp1*. The *ΔAacp1* mutant showed decreased conidiation, more sensitivity to oxidants, reduced secondary metabolites, and virulence.

P450s are playing essential roles in the detoxification of toxic compounds in fungi [7]. Previous research on the tangerine pathotype of the *A. alternata* genome and transcriptome suggested that 13 P450 genes are probably involved in resistance to toxic oxidants. Interestingly, the transcription level of *Aacp1* (AALTg8160) was induced 1.21-fold during H_2_O_2_ stress [21]. To explore whether or not *Aacp1* participates in the detoxification of the ROS mechanism in *A. alternata*, we tested the sensitivity to several ROS-generating compounds between the *ΔAacp1* mutant and WT. In the current study, *ΔAacp1* leads to increased sensitivity to ROS-generating agents including hydrogen peroxide, diethyl maleate, cumyl hydroperoxide, and tert-butyl-hydroxyperoxide in *A. alternata*. The ability of oxidative stress tolerance was restored to the WT level in the *Aacp1-c* strain, suggesting the involvement of *Aacp1* in the oxidative stress response. In *Sporisorium scitamineum*, the MAP kinase Hog1 is involved in the detoxification of the ROS mechanism by regulating the cytochrome P450 encoding gene *SsCPR1* [40]. The deletion mutant *ΔSsCPR1* is hypersensitive to ROS-generating agents, but is not sensitive to osmotic stress [41]. Previous study has shown that Hog1 is also involved in the oxidative stress response and virulence in the tangerine pathotype of *A. alternata* [34]. In the present study, qRT-PCR showed that *Aacp1* was not regulated by *AaHog1*. Therefore, we examined the expression of genes involved in the mechanisms of detoxification of ROS in *A. alternata*, including *Yap1*, *CampA*, *Skn7*, *Hog1*, thioredoxin system (*Tsa1* and *Trr1*), glutaredoxin system (*Gpx3*), and Nox complex (*NoxA*, *NoxB*, and *NoxR*) [29,32,42,43]. Transcriptomic analyses showed these genes had no significant change in *ΔAacp1* compared to the wild-type strain (Table S3). These data indicating the roles of cytochrome P450 in the oxidative stress response have a more complex regulation pattern in the tangerine pathotype *A. alternata.*

Cytochrome P450 monooxygenases are required for conidia production in fungi. In *Magnaporthe oryzae*, MoCYP51A has been shown to be required for conidiation and pathogenicity, but is not required for hyphal growth [11]. Deletion of *Aacp1* resulted in *A. alternata* with a decrease in conidial formation. Despite the conidia being involved in the disease cycle of many plant pathogenic fungi, the molecular mechanisms of sporulation remain unclear [44]. The APSES transcription factor StuA regulates sporulation in many filamentous fungi. Deletion of *StuA* drastically reduced the production of conidia in *A. alternata* [45]. Csn5 is a subunit of the COP9 signalosome, which is essential for conidiation and infection in *A. alternata* [46]. Additionally, several genes including *Slt2*, *VelB*, and *Fus3* have been identified to be essential for the production of conidia in the tangerine pathotype *A. alternata* [34,47,48]. In reference to transcriptome data, the expression of these genes showed no significant changes in *ΔAacp1* (Table S2). The transcriptional levels of conidia-related genes in other plant pathogenic fungi were also examined, including *G alpha*, *PKA*, *Plc*, *Flb*, and *LreA* (Table S2) [44]. However, transcriptome results showed these genes were also not affected in *ΔAacp1*. These data indicate that *Aacp1* may regulate sporulation and mediate a different mechanism in *A. alternata*. Interestingly, RNA-seq and qRT-PCR analyses revealed that the expression of *AaNdo1*, a naphthalene dioxygenase gene, was downregulated by 4.18-fold in *ΔAacp1* compared to the wild-type Z7 strain in the current study. Deletion of *AaNdo1* caused a drastic decrease in conidia production and weakened the infection ability of *A. alternata* on citrus. Naphthalene dioxygenase is one of the key enzymes for the biodegradation of harmful pollutants. Our study has found, for the first time, that naphthalene dioxygenase could affect the formation of conidia in fungi. Overall, these results indicate that *Aacp1* is involved in conidial production partially by regulating the transcription of the *AaNdo1* gene in *A. alternata*.

Cytochrome P450 monooxygenases have been shown to be involved in virulence in various fungi. In *Botrytis cinerea* T4, *CND5* encoding a P450 monooxygenase is essential for the infection ability on tomato leaves [49]. In *S. scitamineum*, deletion of *SsCYP86* resulted in significantly reduced virulence in sugarcane seedlings [40]. Similar to *A. alternata*, pathogenicity tests indicate that *ΔAacp1* greatly reduces the typical necrotic lesion on citrus leaves. P450s are also required for the biosynthesis of secondary metabolites in filamentous fungi [50]. According to KEGG and GO analyses, the transcription levels of secondary metabolism-related genes were broadly regulated in the absence of *Aacp1*, indicating that *Aacp1* also participates in the biosynthesis of secondary metabolites in *A. alternata*. ACT toxin produced by the tangerine pathotype *A. alternata* is a low-molecular-weight secondary metabolite [51]. Previous studies have suggested that ACT toxin plays a critical role in pathogenicity [13]. In the present study, toxicity tests showed that deletion of *Aacp1* in *A. alternata* resulted in the mutant reducing the production of ACT toxin. These results indicate the reduction in virulence of *ΔAacp1* is largely due to the production of less ACT toxin than the WT. However, the roles of cytochrome P450 in ACT toxin production warrant further investigation in the tangerine pathotype *A. alternata.*

Our research has, for the first time, established the biological functions of cytochrome P450 monooxygenase in the tangerine pathotype *A. alternata*. However, the function of *Aacp1* or more cytochrome P450 monooxygenases in *A. alternata* still needs further exploration. The response of *ΔAacp1* to other environmental stresses is still unclear. We need to further determine which genes interact with *Aacp1* in *A. alternata*, to regulate the ACT toxin production and virulence. The molecular mechanism of *AaNdo1* and *Aacp1* also needs further exploration. Moreover, further elucidation of which genes in the host interact with *Aacp1* and inhibitors may provide the potential target sites for application in fungicides.

In conclusion, cytochrome P450 monooxygenase *Aacp1* is required for sporulation, oxidative stress response, ACT toxin production, and pathogenicity in *A. alternata.* Surprisingly, this study further highlights that *Aacp1* regulates transcriptional expression of a naphthalene dioxygenase encoding gene, *AaNdo1*, to participate in sporulation in *A. alternata*. These findings provide a deeper understanding of cytochrome P450 monooxygenase in plant pathogenic fungi.

## Data Availability

Relevant data supporting the findings of this study are available in this article and its Supplementary Information files. The transcriptomic data have been uploaded to the SRA database (accession number SAMN45934042).

## Supplementary Materials

The following supporting information can be downloaded at: https://www.mdpi.com/article/10.3390/microorganisms13020343/s1, Figure S1: Identification and deletion of *Aacp1*; Figure S2: *Aacp1 *is not required for cell-wall integrity and osmotic stress resistance; Figure S3: The expression of *AaNdo1* genes in *ΔAacp1* and identification of *ΔAaNdo1*; Table S1: Oligonucleotide primers used in this study; Table S2: Expression of sporulation-related genes in *A. alternaria*, *A. nidulans*, *N. crassa*, and *Magnaporthe oryzae*; Table S3: Expression of genes associated with detoxification of ROS in *A. alternaria*.

## Appendix A

P450	cytochrome P450 monooxygenase
