# Supplementary material for: A Cytochrome P450 AaCP1 Is Required for Conidiation and Pathogenicity in the Tangerine Pathotype of Alternaria alternata
Source: Microorganisms. 2025 Feb 5;13(2):343. doi: 10.3390/microorganisms13020343 (PMC11858242; doi:10.3390/microorganisms13020343)
Supplement: Supplementary file 1 [file microorganisms-13-00343-s001.zip › microorganisms-3409867-supplementary.pdf]

microorganisms

MDPI

A Cytochrome P450 AaCP1 Is Required for Conidiation and Pathogenicity in the Tangerine Pathotype of *Alternaria alternata*

Huilan Fu [1,*,†], Wenge Li [2,†] and Jintian Tang [2]

[1] College of JunCao Science and Ecology, Fujian Agriculture and Forestry University, Fuzhou 350002, China
[2] Zhejiang Provincial Key Laboratory of Biometrology and Inspection & Quarantine, College of Life Sciences, China Jiliang University, Hangzhou 310018, China; liwenge0130@163.com (W.L.); jintiantang@cjlu.edu.cn (J.T.)
* Correspondence: fuhuilan@fafu.edu.cn; Tel.: +86-18650709337
† These authors contributed equally to this work.

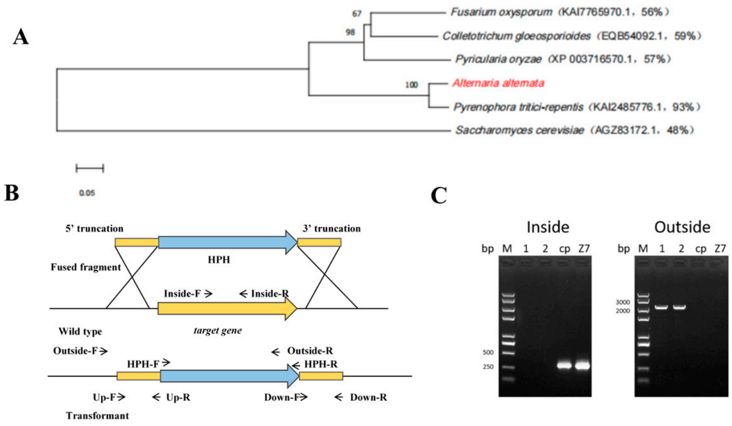

Figure S1. Identification and deletion of *Aacp1*. (A) Phylogenetic tree of CP1 with the homologs from other species were constructed by the MEGA 5.0 program. (B) Schematic illustration of a double joint PCR strategy for disruption of the *Aacp1* gene. (C) Image of DNA fragments amplified from genome DNA of Z7, two transformants, and rescued strain with the primers indicated. Primers Aacp1-inside-F and Aacp1-inside-R were used to examine site-specific integration of HPH within the *Aacp1* allele.

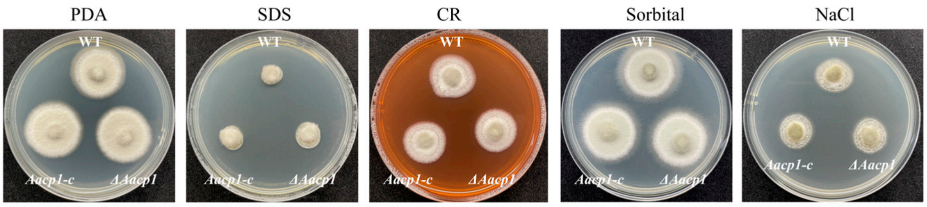

Figure S2. Aacp1 is not required for cell-wall integrity and osmotic stress resistance. Colonies of the wild-type strain Z7, *ΔAacp1*, and *Aacp1-c* on PDA plates containing 100ug/mL Sodium dodecylsulfate (SDS), 100ug/mL Congo red (CR), 100mol/L Sorbital, and 1mol/L NaCl.

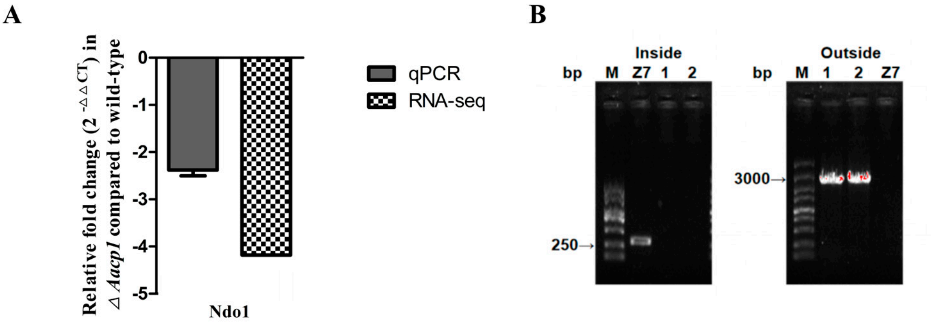

Figure S3. The expression of *AaNdo1* genes in *ΔAacp1* and identification of *ΔAaNdo1*. (A) The relative transcript level of *AaNdo1* was analyzed by RNA-Seq and analyzed by QRT-PCR. The relative expression level from three independent reactions was calculated by a comparative Ct method (ΔΔCT) in relation to the expression of the fungal β-Actin-coding. (B) Image of DNA fragments amplified from genome DNA of wild-type, two transformants, and rescued strain with the primers indicated. Primers AaNdo1-inside-F and AaNdo1-inside-R were used to examine site-specific integration of HPH within the *AaNdo1* allele.

Table S1. Oligonucleotide primers were used in this study.

Primer	sequence
Aacp1-up-F	CGCCTTCAGTGATACGACA
Aacp1-up-R	CTTCTGTCGACTCTAGAGCGGCCGGAAGCCAGCAGGGATAAAA
Aacp1-HYG-F	TTTTATCCCTGCTGGCTTCCGGCCGCTCTAGAGTCGACAGAAG
Aacp1-HYG-R	ACTGTAGGATTGGTCCGTCCTACTCTATTCCTTTGCCCTCGGAC
Aacp1-down-F	GTCCGAGGGCAAAGGAATAGAGTAGGACGGACCAATCCTACAGT
Aacp1-down-R	GCAGTGGGTACAGGTTATG
Aacp1-inside-F	CTTTCCGTCAACACCGCCACT
Aacp1-inside-R	GCGTCGTTCCAGCCACCAT
Aacp1-outside-F	GCTTCCAGTATGGGTTCTC
Aacp1-outside-R	CAGCGTCTCCGACCTGA
CPNeoAacp1-F	AATTCGAGCTCGGTACCCGGGGATCCAGATTCCTCCTTAGCCTTCC
CPNeoAacp1-R	ATGTGTTGACCTCCCGGGATCCTGCTCTAGCCCTAAACTGTA
AaNdo1-up-F	AGCCACAGACTGGTAAGCC
AaNdo1-up-R	CTTCTGTCGACTCTAGAGCGGCCGAAGCCAAAGTAACCAAGGA
AaNdo1-HYG-F	TCCTTGGTTACTTTGGCTTCGGCCGCTCTAGAGTCGACAGAAG
AaNdo1-HYG-R	CTTCACTCGGTTTGTCGATCTACTCTATTCCTTTGCCCTCGGAC
AaNdo1-down-F	GTCCGAGGGCAAAGGAATAGAGTAGTGGAGCAAAGTATGAAGGA
AaNdo1-down-R	ATCGACAAACCGAGTGAAG
AaNdo1-inside-F	CGCTACAATATCGCCAACT
AaNdo1-inside-R	TACCTCAGGCTCCTTCTTT
AaNdo1-outside-F	GTATCTGCGTCATTTCGTC
AaNdo1-outside- R	CAGCGTCTCCGACCTGA

Table S2. Expression of sporulation-related genes in *A.alternaria, A.nidulans, N.crassa, and Magnaporthe oryzae*.

Gene	Accession number	Log2FC	p-value
Fus3	AALT_g9024	-0.289270326	0.372048754337373
Slt2	AALT_g11502	-0.636958117	0.031368316769502
csn5	AALT_g2342	-0.097870957	0.722075493236015
G alpha	AALT_g3129	-0.34733782	0.301243718443892
PKA	AALT_g3695	-0.105594608	0.659186236965413
Plc	AALT_g43	-0.64532152	0.116489276346723
VelB	AALT_g7603	-0.644688012	0.0141972834357854
flbA	AALT_g2799	-0.500173285	0.167214628299046
LreA	AALT_g5872	-0.360556894	0.159330671065718
StuA	AALT_g10904	-0.25740941	0.298340527139219

Table S3. Expression of genes associated with detoxification of ROS in *A.alternaria*.

Gene	Accession number	Log2FC	p-value
noxa	AALT_g5618	0.20337059148601	0.425615153382058
noxr	AALT_g5214	-0.02299043	0.927731717777124
noxb	AALT_g6028	0.285263979654096	0.41673319826931
ap1	AALT_g912	-0.493338595	0.0575883916917177
skn7	AALT_g8622	-0.252138859	0.463781890293631
hog1	AALT_g10096	-0.583438824	0.0276895941208518
fus3	AALT_g9024	-0.289270326	0.372048754337373
campA	AALT_g5219	-0.638415538	0.084980352252923
Trr1	AALT_g7769	-0.209939494	0.497776166194698
Tsa1	AALT_g1812	0.359872962337899	0.278660690098626
Gpx3	AALT_g41	0.317965526012447	0.358202514635029
